# Oral Microbiome in Nonsmoker Patients with Oral Cavity Squamous Cell Carcinoma, Defined by Metagenomic Shotgun Sequencing

**DOI:** 10.3390/cancers14246096

**Published:** 2022-12-11

**Authors:** Ian Ganly, Yuhan Hao, Matthew Rosenthal, Hongmei Wang, Jocelyn Migliacci, Bin Huang, Nora Katabi, Stuart Brown, Yi-Wei Tang, Zhiheng Pei, Liying Yang

**Affiliations:** 1 Head and Neck Service, Department of Surgery, Memorial Sloan Kettering Cancer Center, New York, NY 10065, USA; 2 Department of Pathology, NYU Grossman School of Medicine, New York, NY 10016, USA; 3 Center for Genomics and Systems Biology, Department of Biology, New York University, New York, NY 10003, USA; 4 Department of Laboratory Medicine, Memorial Sloan Kettering Cancer Center, New York, NY 10065, USA; 5 Division of Infectious Diseases, Shenzhen Children’s Hospital, Shenzhen 518026, China; 6 Department of Laboratory Medicine, The First Affiliated Hospital of Sun Yat-sen University, Guangzhou 510080, China; 7 Department of Pathology, Memorial Sloan Kettering Cancer Center, New York, NY 10065, USA; 8 Applied Bioinformatics Laboratories, NYU Grossman School of Medicine, New York, NY 10016, USA; 9 Medical Affairs, Cepheid, Danaher Diagnostic Platform, Shanghai 200335, China; 10 Department of Medicine, NYU Grossman School of Medicine, New York, NY 10016, USA; 11 Pathology and Laboratory Medicine Service, Department of Veterans Affairs New York Harbor Healthcare System, New York, NY 10010, USA

**Keywords:** human papilloma virus, oral cavity squamous cell carcinoma, nonsmokers, whole genome sequencing, bacteria, genes, pathways, microbiome

## Abstract

**Simple Summary:**

Smoking is the commonest cause of oral cavity squamous cell carcinoma (OC-SCC), but the cause of OC-SCC in nonsmokers is unknown. Our primary goal was to use metagenomic shotgun sequencing (MSS) to directly define the taxonomic composition and functional potential of oral metagenome in nonsmokers with OC-SCC. We found three bacterial phyla, one genus, and one species were enriched in OC-SCC while two phyla, five genera, and 18 species were enriched in controls. Pathways related to metabolism of flavin, biotin, thiamine, heme, sugars, fatty acids, peptidoglycans, and tRNA were more abundant in OC-SCC while those related to metabolism of nucleotides and essential amino acids were more abundant in controls. The MSS method achieved similar results to the commonly used 16S rRNA gene survey in compositional differentiation but differed greatly from prediction using 16S rRNA genes in functional differentiation of microbiomes in OC-SCC and controls.

**Abstract:**

**Objectives:** Smoking is the commonest cause of oral cavity squamous cell carcinoma (OC-SCC), but the etiology of OC-SCC in nonsmokers is unknown. Our primary goal was to use metagenomic shotgun sequencing (MSS) to define the taxonomic composition and functional potential of oral metagenome in nonsmokers with OC-SCC. **Methods:** We conducted a case–control study with 42 OC-SCC case and 45 control nonsmokers. MSS was performed on DNA extracted from mouthwash samples. Taxonomic analysis and pathway analysis were done using MetaPhlAn2 and HUMAnN2, respectively. Statistical difference was determined using the Mann–Whitney test controlling false discovery rate. **Results:** There was no significant difference in age, sex, race, or alcohol consumption between OC-SCC and control patients. There was a significant difference in beta diversity between OC-SCC and controls. At the phylum level, *Bacteroidetes* and *Synergistetes* were overly represented in OC-SCC while *Actinobacteria* and *Firmicutes* were overly represented in controls. At the genus level, *Fusobacterium* was overly represented in OC-SCC compared with controls, while *Corynebacterium*, *Streptococcus*, *Actinomyces*, *Cryptobacterium*, and *Selenomonas* were overly represented in controls. Bacterial pathway analysis identified overrepresentation in OC-SCC of pathways related to metabolism of flavin, biotin, thiamin, heme, sugars, fatty acids, peptidoglycans, and tRNA and overrepresentation of nucleotides and essential amino acids in controls. **Conclusions**: The oral microbiome in nonsmoker patients with OC-SCC is significantly different from that of nonsmoker control patients in taxonomic compositions and functional potentials. Our study’s MSS findings matched with previous 16S-based methods in taxonomic differentiation but varied greatly in functional differentiation of microbiomes in OC-SCC and controls.

## 1. Introduction

It is estimated that approximately 54,000 people will be diagnosed with oral and oropharyngeal cancer and 11,230 will die in 2022 in the USA [1]. While the most common risk factors for oral cancer are smoking and alcohol, other factors such as genetic predisposition and periodontal disease are also associated with this disease [2,3,4]. Over the past 45 years there has been a drastic decline in smoking prevalence. However, this has not led to a decline in oral cancer incidence [5,6], which suggests that there has been a change in the epidemiology of this cancer and that other factors play a role in its etiology. For example, some bacteria in the oral microbiome might be responsible. The oral microbiome may cause oral cancer via the production of carcinogenic substances (e.g., cytolethal distending toxin, typhoid toxin, nitrosamine, colibactin) that target DNA and elicit mutations, proinflammatory responses, and direct proliferative effects on cells in oral epithelium [7,8]. Reactive oxygen species produced from bacteria interacting with fibroblasts and immune cells can also cause DNA damage in epithelial cells [9]. 

The 16S rRNA gene sequencing method is the most commonly used technique in microbiome-disease association studies due to its cost-effectiveness and the availability of similar studies for comparisons. To date, there have been seven published studies on the microbiome in oral cavity squamous cell carcinoma (OC-SCC) [10,11,12,13,14,15,16]. Of the 33 genera reported to have an association with OC-SCC, 17 are one-time discoveries not confirmed by other studies and three are controversial associations. The remaining 13 genera are confirmed by at least two studies. While differences in study designs and stringency of statistical analyses may contribute to the lack of confirmation or the controversial association in the majority of these 33 genera, the difference in 16S rRNA gene sequencing methods used may also play a role. For assessing taxon abundance, 16S rRNA gene sequencing carries intrinsic biases introduced by the use of different “universal” primers in amplicon-based PCR amplification for 16S rRNA gene sequencing [17,18]. In addition, the wide range in 16S rRNA gene copy number among genomes causes overestimation of bacteria containing more 16S rRNA genes and underestimation of those containing fewer 16S rRNA genes per bacterial cell [19,20]. 

Determining the functional potential of a bacterial community is an important goal in microbiome studies. Several programs are available to predict functional profiles of microbial communities using 16S rRNA gene surveys and known full genomes [21,22]. In the seven microbiome studies in OC-SCC [10,11,12,13,14,15,16], only three pathways are predicted to be associated with OC-SCC by more than one study [10,13,14,15], two pathways are predicted with controversial associations [12,14,16], while the majority of pathways are one-time discoveries not confirmed by any other study. The low level of agreement between studies may be caused by differences in the 16S rRNA gene survey and reference bacterial genomes used in the prediction, as well as the databases used in pathway classification. 

It is by now quite evident that some bacterial species with indistinguishable 16S rRNA genes may differ greatly in genome compositions. Most of these differences are the result of insertions, deletions, and lateral gene transfer events [23]. For instance, there may be a 25% difference in the genomes of *Vibrio splendidus* strains [24], and up to a 40% difference in the number of genes in their genomes among *Escherichia coli* strains [25,26]. In the *Bacillus cereus* group, *B. cereus*, *B. anthracis*, and *B. thuringiensis* would have been classified as a single species if only based on their very similar 16S rRNA genes as well as highly conserved genomes. The definition of species in this group is based on the presence of large plasmids encoding distinct toxins [27]. The genomic and plasmid differences in the *E. coli* and *B. cereus* groups determine functional potentials and disease-causing capabilities [28,29]. These differences cannot be reliably predicted using 16S rRNA gene survey and reference genomes.

Given these weaknesses of 16S rRNA-based methods, direct sequencing of the entire metagenome by metagenomic shotgun sequencing (MSS) offers a more reliable assessment of taxonomic compositions and functional profiles of a microbiome [30,31,32,33,34,35,36]. However, MSS has not been used in studies of the microbiome in OC-SCC. Our primary goal in this study was to use MSS to differentiate cases and controls of OC-SCC in the functional potential of the microbial communities. Besides controlling for sex, age, and alcohol status, we focused on a population of patients who had either never smoked or who had quit smoking for more than over 5 years to identify the bacteria associated with OC-SCC in the nonsmoking population. Our secondary goal was to use MSS to validate findings from the 16S rRNA gene surveys in the microbiome studies in OC-SCC.

## 2. Methods

We carried out a case–control study with 42 patients with OC-SCC and 45 control subjects with no OC-SCC. Total genomic DNA was extracted from cell pellets of mouthwash samples. Details on subjects, sample collection and processing, data generation and analysis are described below. 

### 2.1. Recruitment of Human Subjects for OC-SCC Cases and Controls

A case–control study was approved by the Institutional Review Board of Memorial Sloan Kettering Cancer Center (MSK) (IRB 15-256) and New York University School of Medicine (i15-00389), as previously described [37]. In brief, we recruited 42 OC-SCC cases and 45 non-OC-SCC controls from MSK. There is no simple mathematic parameter for determining the adequacy of sampling of a complex microbiota. Rather, we empirically estimated the adequacy of sample size based on the findings from our previous study “periodontal pathogens are a risk factor of the oral cavity squamous cell carcinoma” [37]. The previous study used 18 nonsmoker OC-SCC cases and 12 controls while the current study used 42 OC-SCC cases and 45 controls, more than double the cases and triple the controls. Assuming the technical power of the shotgun sequencing is similar to that of the 16S rRNA gene sequencing, the increase in sample size was assumed to be adequate for evaluation of the shotgun sequencing in the differentiation of microbiomes between OC-SCC cases and controls. Patients with OC-SCC in the current study included all patients with OC-SCC except those who were current smokers and those with evidence of recurrence or distant metastases. Non-OC-SCC control patients were patients with either benign or malignant thyroid nodules who had a completely normal head and neck examination, including endoscopy, excluding those who were current smokers. All control patients were either never smokers or had quit smoking. The median number of years since quitting for non-OC-SCC controls was 20 (range 5–63) and for OC-SCC patients was 32 (range 5–57). In patients with oral cancer, OC-SCC was confirmed by histological examination of biopsy specimens. We used histopathological examination to determine the pathological grade and stage of OC-SCC at the time of surgical resection. We recorded demographic and clinical information for each patient.

### 2.2. Detection of Bacterial DNA Sequences in Mouthwash Samples of OC-SCC Patients and Control Patients Using MSS

We used oral rinse specimens from patients with OC-SCC and control patients with no OC-SCC for the detection of bacterial DNA sequences. Oral mouthwash specimens were collected at the time of diagnosis prior to any surgical intervention. This process is demonstrated in Supplementary Figure S1 and detailed below:

a. Mouthwash Sample Collection, Processing, Storage and DNA Extraction 

The participants rinsed their mouths vigorously with 10-mL sterile saline for 30 s and mouthwash was then collected in a 50-mL falcon Conical flask container. After centrifugation at 3120× *g* for 20 min, supernatants were decanted and then the cell pellets were transferred into a 2-mL Eppendorf tube and stored at −80 °C freezer for further study. All oral rinse specimens were taken prior to surgical resection of the OC-SCC. These samples were de-identified and coded. Using the DNeasy Powerlyzer PowerSoil kit (QIAGEN), we successfully extracted DNA from all oral wash samples. DNA yield was measured by the Qubit 2.0 (Invitrogen) with Qubit dsDNA HS Assay kit (ThermoFisher, Waltham, MA, USA). Details can be found in our previous study [37]. 

b. Library Preparation and Sample Sequencing

The DNA fragmentation and shotgun metagenomic library construction and sequencing was carried out at the BGI Americas Corp (Cambridge, MA, USA) using Kapa kit and Illumina HISeq X Ten, with 100 samples pooled into 8 lanes, as previously described [37]. 

c. Raw Sequence Data Quality Control

Raw sequence reads were generated by the Illumina sequencing in fastq format and assigned “Phred scores” describing the base accuracy. FASTQC software was used to evaluate the quality of the raw sequence, and Trimmomatic (v0.36) was used to remove adapters and low-quality bases. Low-quality bases were defined as leading low-quality bases, or N bases, with quality <25; trailing low quality bases, or N bases, with quality <25, scanning the read with a 4-base wide sliding window, and cutting when the average quality per base fell below 25, resulting in reads that were shorter than 50 bases. High-quality reads made up 61.16% of the total reads on average (Minimum: 47.12%; Median: 62.24%; Maximum: 72.69%). By using bowtie2 (v2.2.9) [38], all trimmed reads were aligned to the human genome. Those with a similarity of more than 90% were regarded as human reads.

d. Taxonomic and Functional Pathway Classification

The mapped human reads were filtered out and the non-human reads were analyzed. The non-human reads were then used for taxonomic classification by MetaPhlAn2 (v 2.0) [39], which maps reads to a database of predefined clade-specific marker genes from the phylum to species levels. The downstream analysis eliminated the low abundant taxa (genera < 0.01% and species < 0.001%).

HUMAnN2 (v 0.11.1) [40] was used to analyze the nonhuman reads and identify the abundance of each sample’s gene family and pathway. HUMAnN2 does a translation search to align nonhuman reads to UniRef90 protein clusters (gene families) and maps reads to functionally annotated microbial species genomes [41]. Then, using MinPath, gene families are organized into MetaCyc pathways. Using the “humann2 renorm table” script, we deleted “unintegrated/unmapped/unknown/ungrouped” pathways before computing relative abundance. The low abundant pathways (<0.01%) were removed in the downstream analysis. 

e. Alpha Diversity and Beta Diversity

MetaPhlAn2 taxonomic profiles were used to generate α-Diversity (within-subject diversity) and β-diversity (between-subject diversity). The α-diversity was calculated by function estimate_richness from the package phyloseq [42]. We assessed three α-diversities: Shannon, Simpson, and InvSimpson metrics. The β-diversity was analyzed using weighted and unweighted UniFrac distances matrices [43]. The phylogenetic tree in UniFrac distances were calculated by the curatedMetagenomicData package [44]. Principal coordinate analysis was used for visualization. Nonparametric permutational multivariate analysis of variance (“adonis” function, “vegan” package, R) with 9999 permutations was used to test the association between community-level bacterial compositions.

f. Statistical Analysis 

With the use of the non-parametric Mann–Whitney U test, we first found that there were no statistically significant differences in age, sex, and alcohol uses between the cancer samples and control samples. Thus, in case–control comparison analysis including phylum, genera, species, pathways, alpha diversity, we only use Mann–Whitney U test to identify differential results instead of building regression models to control confounding variables. All statistical tests were two-sided, with a *p* value < 0.05 considered of nominal statistical significance. Target false discovery rates were 20% (q value < 0.2) for taxonomic analysis and 10% (*q* value < 0.1) for pathway analysis. All statistical tests were conducted using R version 4.0.5. 

## 3. Results

### 3.1. Patient and Tumor Characteristics 

The cases and controls did not differ significantly in sex, age, race/ethnicity, alcohol drinking, and cigarette smoking (Table 1). In the 42 patients with OC-SCC, the most common sub-sites were tongue in 24 (57%), floor of mouth in 5 (12%), lower gum in 6 (14.2%), and upper gum in 3 (7.1%) (Table 2). The majority of tumors were stage T1/T2 (32, 78%) and were moderately differentiated (30, 71%). The overall pathological stage was stage I/II 24 (57%) and stage III/IV in 18 (43%) of patients. Fifteen (36%) patients had positive neck node metastases. All cancer patients were treated with primary surgery resection and neck dissection and 18 (43%) required postoperative radiation.

### 3.2. Alpha and Beta Diversity

**Figure 1:**
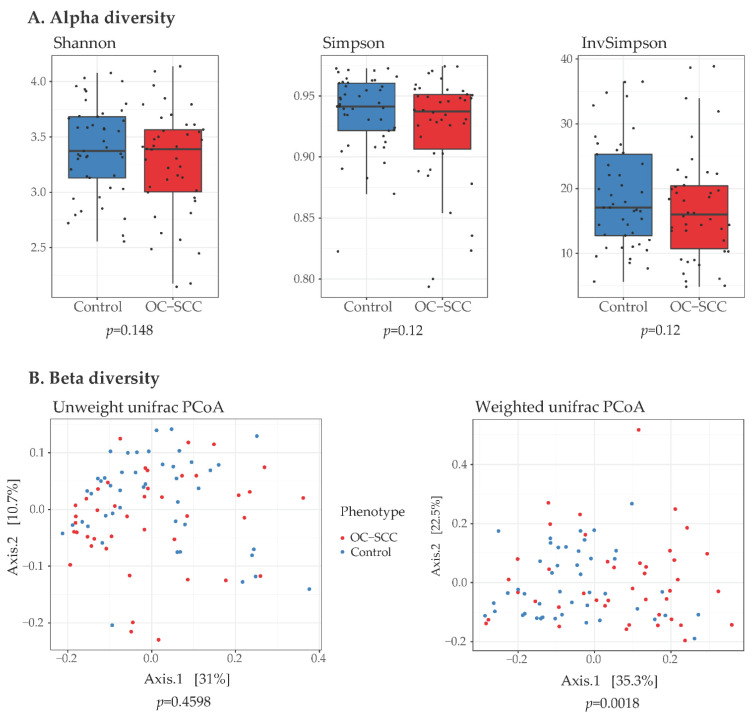
Comparison of alpha and beta diversity between nonsmoking oral cavity squamous cell carcinoma (OC-SCC) patients and non-OC-SCC control patients. (**A**) Boxplot of three types of alpha diversity: Shannon diversity, Simpson index, inverse Simpson index. *P* values were calculated by Mann–Whitney U test. (**B**) Principal coordinate analysis of the global differences of microbiome with unweighted and weighted UniFrac distances matrices. *p* values were calculated by Adonis test with 9999 permutations.

Because within-community diversity (alpha diversity) could alter disease risk in a subject, we compared alpha diversity parameters between OC-SCC cases and controls. There was no significant difference in the bacterial alpha diversity between cases and controls using Shannon (*p* = 0.148), Simpson (*p* = 0.12) and InvSimpson (*p* = 0.12) metrics (Figure 1A). 

To test and characterize between-community diversity (beta diversity), we computed phylogenetic distances between microbial communities in OC-SCC and controls. The analyses of sample-to-sample distances weighted with taxon abundance (weighted beta-diversity) showed that the OC-SCC microbiome clustered separately from that of controls (*p* = 0.0018) (Figure 1B).

### 3.3. Differences in Bacteria Phyla, Genera and Species between Cases and Controls

To identify changes of bacterial abundance in OC-SCC, we next examined the differences in organism abundance between OC-SCC and controls at phylum, genus, and species levels. 

**Figure 2:**
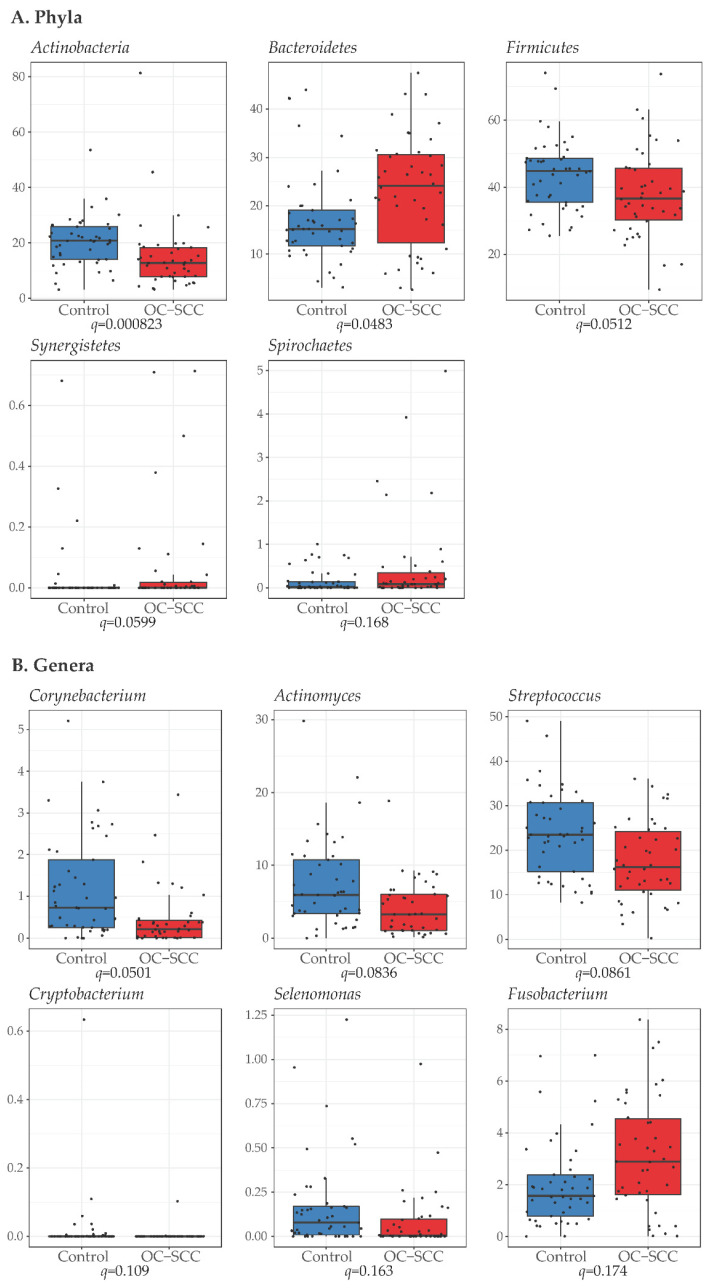
Differential bacterial phyla and genera between nonsmoking OC-SCC patients and non-OC-SCC control patients. The *Y*-axis shows relative abundance in percentage. The difference in the relative abundance of phyla (**A**) and genera (**B**) was evaluated by the Mann–Whitney test and a false discovery rate–adjusted *p* value (*q* value) <0.20 was used as the threshold for significance.

**Figure 3:**
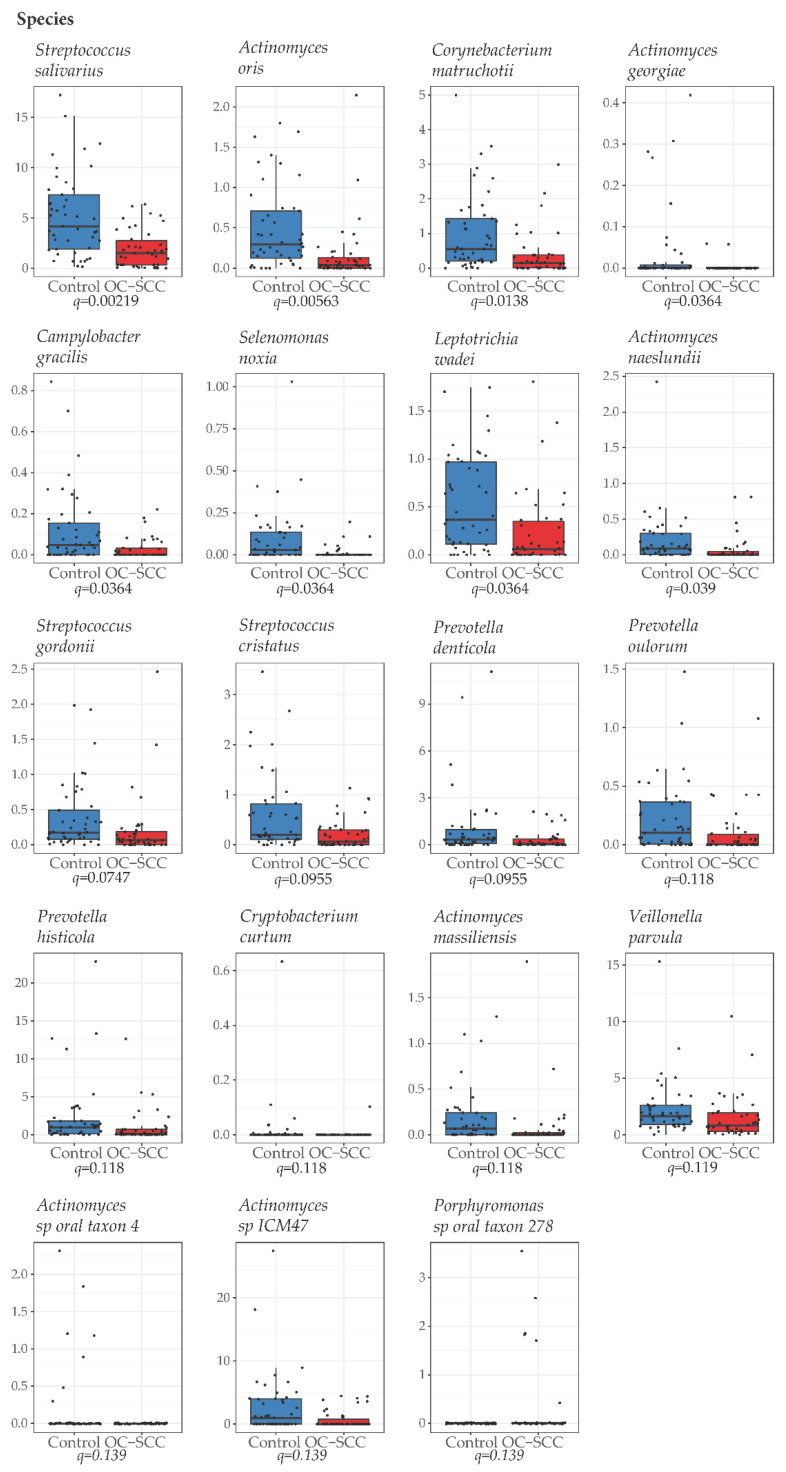
Differential bacterial species between nonsmoking OC-SCC patients and non-OC-SCC control patients. The *Y*-axis shows relative abundance in percentage. The difference in the relative abundance of species was evaluated by the Mann–Whitney test and a false discovery rate–adjusted *p* value (*q* value) <0.20 was used as the threshold for significance.

At the phylum level, *Bacteroidetes* (*q* = 0.0483), *Synergistetes* (*q* = 0.0599), and *Spirochaetes (q* = 0.168) were significantly more abundant in OC-SCC than in controls, while *Actinobacteria* (*q* = 0.0008) and *Firmicutes* (*q* = 0.05) were less abundant in OC-SCC than in controls (Figure 2A, Supplementary Table S1). At the genus level, *Fusobacterium* (*q* = 0.1737) was more abundant in OC-SCC than in controls while *Corynebacterium* (*q* = 0.0501), *Streptococcus* (*q* = 0.0861), *Actinomyces* (*q* = 0.0836), *Cryptobacterium* (*q* = 0.1086), and *Selenomonas* (*q* = 0.1628) were less abundant in OC-SCC than in controls (Figure 2B, Supplementary Table S2). At the species level, the overrepresentation of *Fusobacterium* (*q* = 0.1737) could not be traced to any species in this genus although *Porphyromonas* sp oral taxon 278 (*q* = 0.1386) was found to be more abundant in OC-SCC than in controls. For genera underrepresented in OC-SCC, species-level analysis found that *Corynebacterium* was represented by *Corynebacterium matruchoat* (*q* = 0.0138), *Streptococcus* by three *Streptococcus* species (*salivarius*, *q* = 0.0022; *gordonii*, q = 0.074; *cristatus*, *q* = 0.095), *Actinomyces by* six *Actinomyces* species (*oris*, *q* = 0.0056; *georgiae*, *q* = 0.0364; *naeslundi*, *q* = 0.039; *massiliensis*, *q* = 0.1183; oral_taxon_448, *q* = 0.1386; ICM47, *q* = 0.1386), *Cryptobacterium* by *Cryptobacterium curtum* (*q* = 0.1183), and *Selenomonas* by *Selenomonas noxia* (*q* = 0.0364). Additionally, six species were also found to be less abundant in OC-SCC than in controls, including three *Prevotella species* (*denticola*. *q* = 0.095; *oulorum*, *q* = 0.1183; *histicola*, *q* = 0.1183), *Campylobacter gracilis* (*q* = 0.0364), *Leptotrichia wadei* (*q* = 0.0364), and *Veillonella parvula* (*q* = 0.1193) (Figure 3, Supplementary Table S3). 

### 3.4. Functional Prediction of Oral Microbiome Related to the Development of Oral Cancer 

To go beyond taxonomic description of the oral microbiome, we explored functional differences between OC-SCC cases and controls using HUMAnN2 (v 0.11.1) [40]. Pathway analysis showed significant differences (*q* < 0.01) in 37 pathways. Of the 37 pathways, 15 were more abundant while 22 were less abundant in OC-SCC cases than in controls (Table 3). Specifically, in vitamin biosynthesis, pathways related to the biosynthesis of flavin, biotin, and thiamin (n = 8) were more abundant while those related to folate biosynthesis (n = 2) were less abundant in OC-SCC than in controls. Pathways related to biosynthesis of heme (n = 2), sugars (n = 2), fatty acids (n = 1), and cell wall (n = 2), and tRNA charging (n = 1) were more abundant OC-SCC. In contrast, pathways related biosynthesis of nucleotides (n = 9) and amino acids (n = 7) were less abundant in OC-SCC. In the three pathways related to fermentation, homolactic fermentation was more abundant while pyruvate fermentation and anaerobic energy metabolism were less abundant in OC-SCC than in controls.

## 4. Discussion

Smoking and heavy alcohol consumption have long been identified as risk factors for oral cancer [2]. However, even with the steady drop in population-wide cigarette use and alcohol consumption over the past 40 years, the incidence of oral cancer has not declined [5,6], resulting in a growing number of patients who have oral cancer but do not smoke or drink alcohol [5,6]. These patients tend to be younger and are more likely to be female. It is not clear why they develop oral cancer. To better understand the differences in this group of patients with oral cancer, we performed the first MSS in OC-SCC to differentiate cases and controls based on the functional potential and taxonomic compositions of the oral microbial communities. Despite being more costly than 16S rRNA-based methods, MSS can directly measure a metagenome by unbiased-sequencing of all DNA fragments in a sample rather than indirect inference of the metagenome based on a single marker gene. Our study included 42 oral cancer patients and 45 non-OC-SCC control patients. The OC-SCC patients and control patients were all nonsmokers and well matched in terms of age, sex, and alcohol status. We have shown that nonsmoking OC-SCC patients have a significantly different bacterial microbiome compared with a similar cohort of control patients who do not smoke.

In the previous seven 16S rRNA gene-based studies of microbiome in OC-SCC [10,11,12,13,14,15,16], six studies included both smokers and nonsmokers in the OC-SCC group [10,11,12,13,15,16] and one study excluded current smokers from both cases and controls [14]. The most striking overrepresentation of genus in controls is *Streptococcus* in six of the seven studies (6/7) [10,11,12,14,15,16], followed by *Actinomyces* (3/7) [11,15,16], *Rothia* (3/7) [10,11,16], *Veillonella* (3/7) [10,13,16], and *Haemophilus* (2/7) [13,15]. In contrast, the most striking overrepresentation of genera in OC-SCC are *Campylobacter* [10,11,12,13,16] and *Fusobacterium* [10,12,14,15,16] in five of the seven studies (5/7), followed by *Peptostreptococcus* (4/7) [11,12,13,16], *Catonella* (3/7) [11,13,16], *Alloprevotella* (2/7) [14,16], *Filifactor* (2/7) [13,16], *Parvimonas* (2/7) [11,16], and *Selenomonas* (2/7) [10,16]. The most notable controversial genera are the overrepresentation of *Prevotella* (4/7) [10,11,12,14] and *Capnocytophaga* (3/7) [11,12,16] in OC-SCC in four and three of the seven studies, respectively while opposite findings are reported in one of the seven studies [13]. On the other hand, *Granullicatella* are overrepresented in controls in two of the seven studies [13,16] and overrepresented in OC-SCC in one study [10]. In the present study, MSS confirmed the overrepresentation of *Fusobacterium* and underrepresentation of *Streptococcus* and *Actinomyces* in OC-SCC. *Selenomonas* was underrepresented in contrast to its overrepresentation in OC-SCC in the 16S rRNA-based study. Our new finding is the underrepresentation of *Cryptobacterium* in OC-SCC compared with controls. *Cryptobacterium* is a genus under the phylum *Actinomycetota*. Because of its phylogenetic similarity with *Actinomyces*, it is not surprising to find a similar association with controls. The underrepresentation of *Cryptobacterium* can be traced down to *Cryptobacterium curtum*. *C. curtum* was recently identified and the only known species in *Cryptobacterium* [45]. Similar to *Actinomyces*, *C. curtum* is a Gram-positive anaerobic rod. While the type strains of *C. curtum* were isolated from patients with necrotic dental pulps, root canals, dental abscess, and halitosis, its associations with dental or periodontal diseases have not been established [45,46]. 

For OC-SCC in nonsmokers, we have previously reported a 16S rRNA-based study of the oral microbiome in 18 OC-SCC, 8 premalignant lesions, and 12 control patients and showed a progressive enrichment of periodontal pathogens *Fusobacterium*, *Prevotella*, and *Alloprevotella* and a depletion in *Streptococcus* in nonsmoking patients [14]. Our MSS findings of underrepresentation of *Streptococcus* and overrepresentation of *Fusobacterium* in OC-SCC are in line with the previous findings in the 16S rRNA-based study. Although in our MSS study, the previously found overrepresentation of genera *Prevotella* and *Alloprevotella* in OC-SCC were not evident, at the phylum level above these genera, we found a compatible overrepresentation of *Bacteroidetes* in OC-SCC. In addition, we found underrepresentation of *Corynebacterium* and *Actinomyces* in OC-SCC cases. 

These observations are highly relevant because they now provide evidence that certain bacteria are consistently associated with OC-SCC when surveyed with either 16S gene amplification-based sequencing or MSS. The loss of *Streptococcus* genus is significant as *Streptococcus* species have been shown to impair *Fusobacterium nucleatum*-induced inflammation in oral epithelial cells [47]. A loss of *Streptococcus* species would therefore promote the inflammation associated with *Fusobacterium nucleatum*. The decreases in *Streptococcus* genera and species have been reported in esophageal adenocarcinoma and gastric cancer [48,49]. 

The presence of *Fusobacterium nucleatum* has been shown to protect tumor cells from immune cell attack [50] and stimulate oral cancer development by engagement with the oral epithelium via Toll-like receptors [51]. *Fusobacterium nucleatum* is now recognized as a significant contributor to colorectal cancer [52]. Landmark publications in 2012 reported *Fusobacterium nucleatum* as being prevalent in colorectal cancer [53,54]. It is found in abundance in colorectal cancer tissue, as are high levels of the fusobacterium adhesion A (FadA) molecule, which activates oncogenic signaling to promote colorectal cancer [50]. Other studies reporting the relevance of *Fusobacterium nucleatum* in oral cancer by Binder-Gallimidi showed coinfection of *Fusobacterium nucleatum* with *porphyromonas gingivalis* was found to promote oral cancer by regulating the TLR2-OL6-STAT3 axis in a chemically induced tongue cancer mouse model [51,53]. In addition, a recent report by Geng et al. [55] reported that *Fusobacterium nucleatum* promotes oral cancer by causing DNA double strand breaks via the Ky70/p53 pathway. Previous studies have also shown that DNA damage and repair are affected by other bacteria such as *Helicobacter pylori* or pneumococci [56,57]. A comprehensive review of recent studies by McIlvanna showed *F. nucleatum* can promote cancer through several mechanisms including activation of cell proliferation, promotion of cellular invasion, induction of chronic inflammation, and immune evasion [58]. 

In the previous seven microbiome studies in OC-SCC [10,11,12,13,14,15,16], numerous pathways are predicted based on the 16S rRNA marker gene to have associations with OC-SCC but only a few are confirmed by more than one study. Pathways overrepresented in OC-SCC include lipopolysaccharide biosynthesis (reported in 3 of the 8 studies) [10,13,14], norspermidine biosynthesis pathway (2/8) [13,14], and oxidative phosphorylation and carbon fixation (2/8) [14,15]. However, pathways related to terpenoid biosynthesis and polyketide biosynthesis are overrepresented in two studies and underrepresented in one study [12,14,16]. Using MSS, we found overrepresentation in OC-SCC of pathways related to metabolism of flavin, biotin, thiamine, heme, sugars, fatty acids, peptidoglycans, and tRNA and overrepresentation of nucleotides and essential amino acids in controls. A previous study found that microbial pathway modules associated with metabolism of flavin, biotin, and heme have proliferative activities on cells. Biotin is an essential nutrient belonging to the vitamin B complex. In human cells, biotin-dependent carboxylases catalyze key reactions in gluconeogenesis, fatty acid synthesis, and amino acid catabolism [59]. With regard to flavin biosynthesis, it has been reported that riboflavin at high doses might promote lung cancer progression [60]. Heme is an essential cofactor for enzymes of the electron transport chain. It plays a role in the generation of ATP in oxidative phosphorylation and is therefore essential for cell proliferation [61]. All of these small molecule metabolites produced by the tumor microbiome can easily diffuse into tissues via transporters or free diffusion and then alter the signaling pathways of cancer and immune cells. Hence, targeting the oral tumor microbiome metabolism could offer a novel method of developing a new treatment for oral cancer [62]. The negative association of bacterial biosynthesis of essential amino acids and nucleotides as well as folate suggests abundant availability—potentially provided by foods or degradation of necrotic tumor tissue—of these molecules in the tumor microenvironment that allows bacteria deficient in these pathways to grow. 

Compared with our previous study of the microbiome in nonsmokers with OC-SCC [14], the current MSS method identified 37 differential pathways, which is far fewer than the 102 pathways predicted using the 16S rRNA gene survey. Only 10 pathways were reported by both studies. Of the 10 pathways, four showed concordant changes, with pathways for 8-amino-7-oxononanoate biosynthesis I and tRNA charging overrepresented in OC-SCC and pathways for pyrimidine nucleobase salvage and pyrimidine deoxyribonucleotide phosphorylation overrepresented in controls. Six pathways showed discordant changes, with pathways for peptidoglycan biosynthesis, N10-formyl-tetrahydrofolate biosynthesis, peptidoglycan maturation (meso-diaminopimelate containing), aspartate superpathway, L-lysine biosynthesis II, and purine nucleotides de novo biosynthesis I overrepresented in controls in the MSS study but in OC-SCC in the prediction using 16S rRNA gene survey. 

Although our findings in changes of taxonomic composition of the oral microbiome in OC-SCC are supported by previous studies, the unique findings in the functional potential of the microbiome could be due to differences in the study cohort in addition to the MSS method used. We did not have details on the immune, nutrition, and dental status of the patients, which could impact the oral microbiome and introduce the differences we observed. The differences between OC-SCC and non-OC-SCC controls may not reflect the difference between OC-SCC patients and healthy subjects because the control subjects had benign thyroid lesions, which may have an unknown impact on the oral microbiome. Additionally, our population of patients was predominantly white and all subjects were from the USA, leaving the possibility that the oral microbiome may differ based on ethnicity as well as geography. Further research in different geographic and ethnic populations is therefore needed. 

## 5. Conclusions

Our study indicates that MSS is in general agreement with 16S-based methods in taxonomic differentiation of microbiomes in OC-SCC and controls. We found that the taxonomic composition of the oral microbiome in patients with OC-SCC is similarly altered in nonsmokers and smokers. As such, MSS can be used independently or as a method to validate findings from 16S rRNA gene surveys in studies of the microbiome in OC-SCC. In contrast, our study reveals poor agreement between MSS and 16S-based methods in functional differentiation of microbiomes in OC-SCC and controls. Future studies should directly compare both methods using the same set of OC-SCC cases and controls to minimize any discrepancy caused by differences in study design, specimens, pathway assignment, and statistical stringency. If substantial differences still exist, future research will need to focus on identifying the sources of artificial differences. 

## Figures and Tables

**Table 1:** Patient characteristics of nonsmoking OC-SCC patients and controls.

Characteristics	OC-SCC (*n* = 42)	Controls (*n* = 45)	*p* Value
Sex (%)			
Male	19 (45%)	24 (53%)	0.5
Female	23 (55%)	21 (47%)	
Age (mean ± SD)			0.7
	63 ± 13	63 ± 11	
Race (%)			
White	34 (81%)	37 (82%)	0.9
Others	8 (19%)	8 (18%)	
Alcohol drinking (%)			0.3
Never/social drinking	12 (29%)	15 (33%)	
Quit	3 (7.1%)	0 (0%)	
Active	27 (64%)	30 (67%)	
Social/mild	20 (71%)	21 (70%)	
Moderate	4 (14%)	5 (17%)	>0.9
Heavy	4 (14%)	4 (13%)	
Smoking (%)			>0.9
Never	22 (52%)	24 (53%)	
Quit	20 (48%)	21 (47%)	

**Table 2:** Pathology characteristics of OC-SCC cases.

Characteristic	No (%)
Tumor subsite	
Tongue	24 (57%)
Floor of mouth	5 (12%)
Upper gum	3 (7.2%)
Lower gum	6 (14%)
Buccal	2 (4.8%)
Retromolar trigone	2 (4.8%)
Lip	0
Treatment	
Surgery alone	24 (57%)
Surgery + postop radiation	18 (43%)
Tumor size (mm)	
1–10	11 (26%)
11–20	14 (33%)
21–30	8 (19%)
31–40	5 (12%)
41–50	4 (9.5%)
Pathology T stage	
T1	21 (51%)
T2	11 (27%)
T3	2 (4.9%)
T4	7 (17%)
Pathology N stage	
N0/Nx	27 (64%)
N+	15 (35.7%)
Overall pathological stage	
1	20 (47%)
2	4 (9.3%)
3	6 (14%)
4	12 (28%)
Tumor grade	
Well differentiated	10 (24%)
Moderately differentiated	30 (71%)
Poorly differentiated	2 (4.8%)

**Table 3:** Significant changes in 37 microbial pathways in oral microbiome in nonsmoking OC-SCC patients.

Class	Pathway	Control *	Cancer *	Fold Change	*q* Value
Vitamin	PWY-6519: 8-amino-7-oxononanoate biosynthesis I	11	17	1.530	0.028
	BIOTIN-BIOSYNTHESIS-PWY: biotin biosynthesis I	12	18	1.491	0.028
	PWY-7539: 6-hydroxymethyl-dihydropterin diphosphate biosynthesis III	28	37	1.293	0.057
	RIBOSYN2-PWY: flavin biosynthesis I bacteria and plants	29	36	1.243	0.023
	THISYNARA-PWY: superpathway of thiamin diphosphate biosynthesis III	29	34	1.202	0.059
	PWY-6168: flavin biosynthesis III	30	35	1.194	0.059
	PWY-6147: 6-hydroxymethyl-dihydropterin diphosphate biosynthesis I	65	76	1.166	0.031
	PWY-6897: thiamin salvage II	39	43	1.112	0.094
	PWY-3841: folate transformations II	66	58	0.874	0.059
	1CMET2-PWY: N10-formyl-tetrahydrofolate biosynthesis	57	50	0.873	0.059
Heme	HEME-BIOSYNTHESIS-II: heme biosynthesis I aerobic	23	30	1.322	0.028
	PWY-5918: heme biosynthesis from glutamate	33	40	1.216	0.059
Nucleotide	PWY-7228: guanosine nucleotides de novo biosynthesis I	85	80	0.947	0.077
	PWY-6126: adenosine nucleotides de novo biosynthesis II	98	92	0.946	0.098
	PWY-841: purine nucleotides de novo biosynthesis I	71	66	0.938	0.094
	PWY-6125: guanosine nucleotides de novo biosynthesis II	81	76	0.938	0.077
	PWY-7208: pyrimidine nucleobases salvage	79	73	0.923	0.059
	PWY-7197: pyrimidine deoxyribonucleotide phosphorylation	66	61	0.920	0.077
	PWY-7220: adenosine deoxyribonucleotides de novo biosynthesis II	72	66	0.917	0.059
	PWY-7222: guanosine deoxyribonucleotides de novo biosynthesis II	72	66	0.917	0.059
	PWY0-1296: purine ribonucleosides degradation	67	57	0.850	0.057
tRNA	TRNA-CHARGING-PWY: tRNA charging	34	39	1.152	0.059
Amino acid	ILEUSYN-PWY: L-isoleucine biosynthesis I from threonine	93	87	0.937	0.094
	VALSYN-PWY: L-valine biosynthesis	93	87	0.937	0.094
	PWY-2941: L-lysine biosynthesis II	59	54	0.914	0.090
	PWY-6936: seleno-amino acid biosynthesis	76	69	0.907	0.059
	PWY0-781: aspartate superpathway	35	32	0.895	0.065
	P4-PWY: L-lysine L-threonine and L-methionine biosynthesis I	42	37	0.895	0.094
	PWY-5347: L-methionine biosynthesis transsulfuration	52	43	0.831	0.023
Sugar	DTDPRHAMSYN-PWY: dTDP-L-rhamnose biosynthesis I	43	49	1.148	0.094
	CALVIN-PWY: Calvin-Benson-Bassham cycle	49	55	1.120	0.038
Fatty acid	PWY-5973: cis-vaccenate biosynthesis	54	58	1.087	0.094
Fermentation	ANAEROFRUCAT-PWY: homolactic fermentation	38	41	1.096	0.059
	PWY-7111: pyruvate fermentation to isobutanol engineered	94	87	0.932	0.077
	PWY-7383: anaerobic energy metabolism invertebrates cytosol	27	22	0.849	0.059
Cell wall	PWY0-1586: peptidoglycan maturation	20	17	0.848	0.063
	PWY-6471: peptidoglycan biosynthesis IV Enterococcus faecium	21	16	0.742	0.028

* Reads per 100,000 reads.

## Data Availability

The data presented in this study is available on request from the corresponding author.

## Supplementary Materials

The following supporting information can be downloaded at: https://www.mdpi.com/article/10.3390/cancers14246096/s1, Figure S1: Flowchart of sample and data processing of oral microbiome in OC-SCC; Table S1: Changes in phylum abundance in oral microbiome in nonsmoking OC-SCC patient; Table S2: Changes in genus abundance in oral microbiome in OC-SCC; Table S3: Changes in species abundance in oral microbiome in OC-SCC.
